# HBx Protein Promotes Oval Cell Proliferation by Up-Regulation of Cyclin D1 via Activation of the MEK/ERK and PI3K/Akt Pathways

**DOI:** 10.3390/ijms15033507

**Published:** 2014-02-26

**Authors:** Heng-Yi Wang, Sheng-Li Yang, Hui-Fang Liang, Chang-Hai Li

**Affiliations:** 1 Department of Surgery, the First Affiliated Hospital of Anhui Medical University, Hefei 230022, China; 2 Hepatic Surgery Center, Tongji Hospital, Tongji Medical College, Huazhong Science and Technology University, Wuhan 430030, China; E-Mail: lichvip@126.com; 3 Department of General Surgery, Liyuan Hospital, Tongji Medical College, Huazhong Science and Technology University, Wuhan 430077, China; E-Mail: jixufendou2013@gmail.com

**Keywords:** HBx, oval cell, proliferation, cyclin D1, ERK, Akt

## Abstract

Growing evidence has shown that hepatic oval cells, also named liver progenitor cells, play an important role in the process of liver regeneration in various liver diseases. Oval cell proliferation has been reported in hepatitis B virus (HBV)-associated hepatocellular carcinoma (HCC) and chronic liver disease. Studies have found expression of HBV surface and core antigens in oval cells in the livers of patients with HCC, suggesting that HBV infection of oval cells could be a mechanism of human hepatocarcinogenesis. In addition, there is evidence of multiplication of HBV in oval cell culture. However, little research has been performed to explore the role of HBV-encoded proteins in the proliferation of hepatic oval cells. Previously, we successfully transfected the *HBV x* (*HBx*) gene, one of the four genes in the HBV genome, into a rat LE/6 oval cell line. In this study, we tested whether or not the transfected *HBx* gene could affect oval cell proliferation *in vitro*. Our results show that overexpression of *HBx* promotes the proliferation of oval cells and increases cyclin D1 expression, assessed at both the mRNA and protein levels. We also found that HBx activated the PI-3K/Akt and MEK/ERK1/2 pathways in HBx-transfected oval cells. Furthermore, the HBx-induced increases in cyclin D1 expression and oval cell proliferation were completely abolished by treatment with either MEK inhibitor PD184352 or PI-3K inhibitor LY294002. These results demonstrated that HBx has the ability to promote oval cell proliferation *in vitro*, and its stimulatory effects on cell proliferation and expression of cyclin D1 depend on the activation of the MEK/ERK and PI3K/Akt signaling pathways in cultured oval cells.

## 1. Introduction

Hepatic oval cells are a subpopulation of liver cells that are quiescent under normal conditions and exist in low numbers around the periportal region. When hepatocytes are not able to divide and replace damaged tissues, oval cells are activated for proliferation [1,2]. It is well known that oval cell proliferation is significantly enhanced in serious liver injuries caused by drugs, viruses, and toxins [3,4]. In addition, the magnitude of oval cell proliferation correlates with the severity of parenchymal inflammation in human and animal models [5]. Previous studies focus primarily on the mitogenic role of external cytokines and growth factors derived from inflammatory cells, including Kupffer cells, lymphocytes, natural killer T cells, natural killer cells, and hepatocytes. These cells are primarily responsible for the activation and expansion of the oval cell population *in vivo* [6–8].

The molecular networks for regulation of oval cell activation and proliferation have been investigated *in vitro*. There is evidence that interleukin-6 (IL-6) and interferon-γ (IFN-γ) induce oval cell proliferation by activating signal transducer and activator of transcription 3 (STAT3) *in vitro* [9]. Studies have shown that tumor necrosis factor alpha (TNF-α) also stimulates proliferation of LE/6 oval cells and that LE/6 cells are less responsive to suppression by transforming growth factor beta (TGF-β) [9,10].

In humans, oval cell activation has been reported in hepatitis B virus (HBV)-associated hepatocellular carcinoma (HCC) and chronic liver disease [1,3,4]. The *HBV x* (*HBx*) gene, one of the four genes in the HBV genome, was detected in HBV-associated HCC liver tissues at a high rate [11–13]. Previous studies have found expression of HBV surface and core antigens in oval cells of livers in patients with HCC, which also suggested that HBV infection of oval cells could be a mechanism of human hepatocarcinogenesis [14]. There is also evidence of HBV multiplication in oval cell culture [15]. These facts indicate that HBV infection occurs in oval cells in humans and in cell culture, and that HBV-encoded proteins may affect the proliferation of oval cells.

The HBV-encoded protein, HBx, has been shown to promote cell proliferation by activating intracellular signaling transduction cascades, including the Ras/Raf/MEK/ERK, PI-3K-Akt/PKB, c-Jun *N*-terminal kinase (JNK), and Janus kinase (Jak)/STAT pathways [16–19]. The extracellular signal-regulated kinase (ERK) is a subfamily member of mitogen-activated protein kinases (MAPKs), which are activated by an upstream kinase called MAPK/ERK kinase (MEK). The ERK pathway mediates a number of cellular fates, including cell growth, proliferation, and survival [20,21]. The serine/threonine kinase Akt, also known as protein kinase B (PKB), plays a pivotal role in cell proliferation, differentiation, and survival and is activated by a phosphoinositide 3-kinase (PI3K)-dependent signaling pathway [22].

Previously, we established a hepatic oval cell line (LE/6) that stably expressed HBx protein [23]. In this study, we tested whether the HBx protein affects oval cell proliferation *in vitro*. Moreover, we further investigated the role of Akt and ERK in up-regulation of cyclin D1 protein expression. Our data shows that HBx-induced up-regulation of cyclin D1 expression and cell proliferation were severely inhibited by PI3K and MEK inhibitors in oval cells. These results indicate that HBx protein promotes oval cell proliferation by up-regulation of cyclin D1 via activation of the MEK/ERK and PI3K/Akt signaling pathways.

## 2. Results and Discussion

### 2.1. Expression of HBx in LE/6 Cells

**Figure 1.**
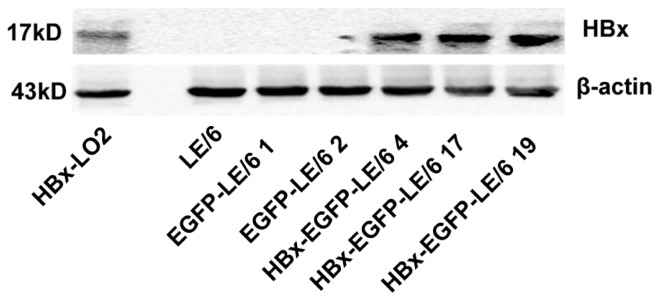
Western blotting analysis showed that HBx-transfected clones 4, 17, and 19 strongly expressed HBx protein. Non-transfected LE/6 and mock-transfected enhanced green fluorescent protein (EGFP)-LE/6 clones 1 and 2 were used as negative controls that did not express HBx protein. HBx transfected L02 cells served as a positive control for transfection efficiency.

We confirmed the expression of HBx protein in HBx-transfected clones by western blotting (Figure 1). These results indicated that the HBx protein was overexpressed in HBx-transfected oval cells.

### 2.2. HBx Promotes Proliferation of LE/6 Cells

**Figure 2.**
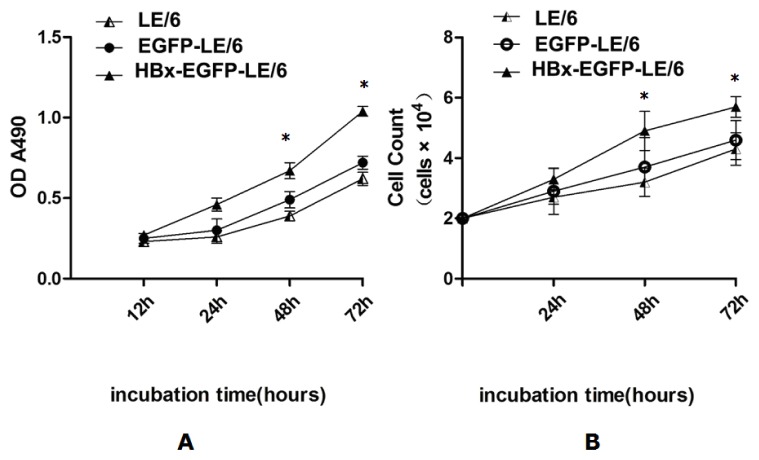
Cells were uniformly seeded in each well of 96-well plates and grown in DMEM supplemented with 10% FBS. After 24 h, the media was removed and replaced with fresh media. After incubation for an additional 12, 24, 48, and 72 h, cell proliferation was assessed by MTT (**A**) and cell count (**B**) assays. Results from both assays showed that HBx expression led to a significant increase in oval cell proliferation compared with controls at the 48 and 72 h time-points. An asterisk * indicates *p* < 0.05 *vs.* LE/6 and EGFP-LE/6 controls. The data shown represent the mean ± standard deviation (SD) of three independent experiments.

A cell count assay and an MTT (3-[4,5-dimethylthiazol-2-yl]-2,5 diphenyl tetrazolium bromide) assay were performed to evaluate whether HBx had an effect on oval cell proliferation. The results showed that HBx significantly increased oval cell proliferation compared with controls (Figure 2A,B).

### 2.3. Expression of Modulators in Cell Cycle Progression in HBx Overexpressing Cells

**Figure 3.**
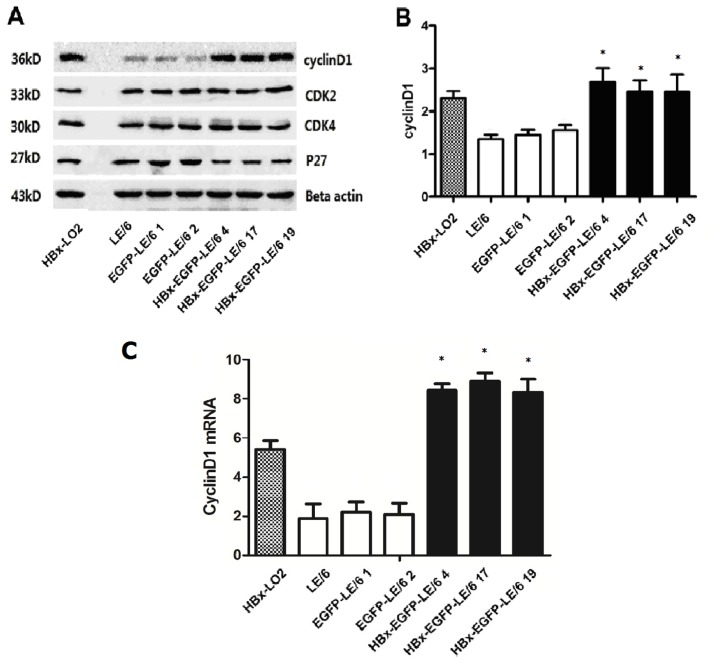
(**A**) Western blotting indicated that overexpression of HBx significantly increased cyclin D1 protein expression in oval cells. In contrast, expression of p27, CDK2 and CDK4 proteins were unaltered; (**B**) The bands of cyclin D1 shown in (**A**) were quantified and the band intensities are shown graphically; (**C**) qPCR results indicated that mRNA levels of cyclin D1 were remarkably increased in HBx-transfected oval cells as compared with controls. An asterisk * indicates *p* < 0.05 *vs.* LE/6 and EGFP-LE/6 controls. The data shown represent the mean ± SD of three independent experiments.

To explore the mechanism of HBx-induced cell proliferation in oval cells, intracellular levels of cell cycle modulators were determined by western blotting. In the HBx-transfected oval cells, cyclin D1 protein expression was increased as compared to that in controls (Figure 3A,B). The overexpression of HBx had no effect on p27, cyclin-dependent kinase 2 (CDK2), or cyclin-dependent kinase 4 (CDK4) protein expression (Figure 3A). Quantitative real-time PCR (qPCR) data also revealed an increase in cyclin D1 mRNA levels in HBx-transfected oval cells (Figure 3C). These data suggest that up-regulated cyclin D1 expression might play a key role in HBx-induced oval cell proliferation.

### 2.4. Up-Regulation of Cyclin D1 by HBx in Oval Cells Is Dependent of the Activation of MEK/ERK and PI-3K/Akt Signaling Pathways

**Figure 4.**
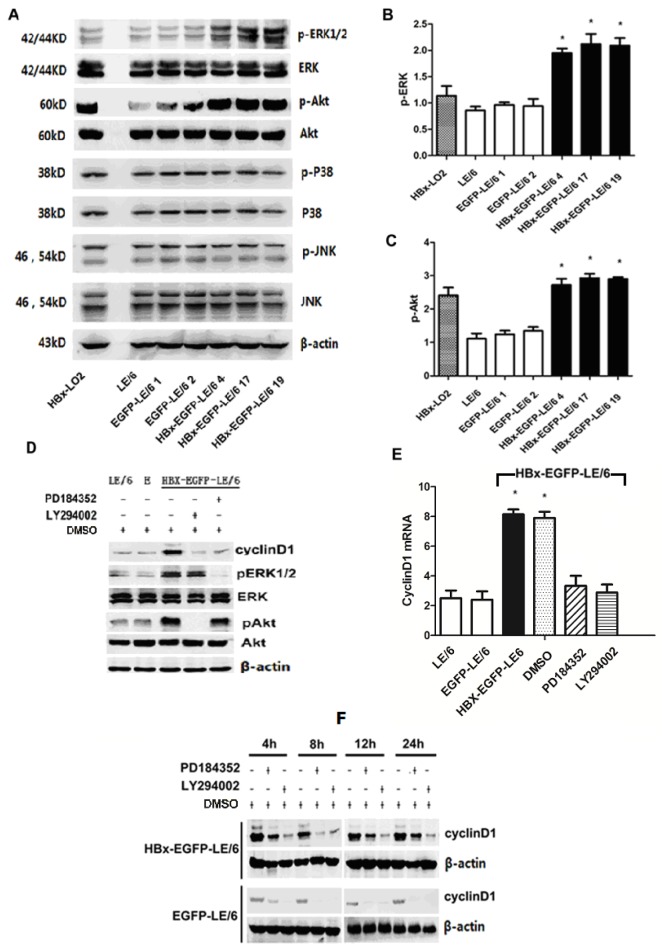
(**A**) Western blotting results indicated that the levels of phosphorylated Akt and ERK1/2 were elevated in HBx-transfected cells compared to the levels in non-transfected and mock-transfected controls; however, pJNK and pp38 levels remained unchanged. Quantitation of the band intensities of pERK (**B**) and pAkt (**C**) is shown. (**D**) HBx-EGFP-LE/6, EGFP-LE/6, and LE/6 oval cells were treated with PD184352 (5 μM), LY294002 (25 μM), or vehicle control (DMSO) for 24 h and lysed for western blotting or qPCR (**E**) analysis. PD184352 or LY294002 treatment blocked ERK and Akt activation, leading to reduced expression of cyclin D1 at both the mRNA and protein levels. (**F**) HBx-EGFP-LE/6, EGFP-LE/6, and LE/6 oval cells were treated with PD184352, LY294002, or a DMSO control for 24 h. Cyclin D1 expression in each treatment group was analyzed at the indicated time-points. Treatment with either PD184352 or LY294002 inhibited cyclin D1 expression at the protein level. The data shown represent three independent experiments and an asterisk * indicates a *p*-value < 0.05.

We performed western blotting to confirm whether Akt/phospho-Akt (pAkt), ERK/phospho-ERK (pERK), JNK/phospho-JNK (pJNK), and p38/phospho-p38 (pp38) were involved in HBx-induced proliferation. Transfection of HBx significantly increased the activation of pERK (Figure 4A,B) and pAkt (Figure 4A,C); however, the total amounts of Akt, ERK, JNK/pJNK, and p38/pp38 remained unchanged in HBx-transfected oval cells (Figure 4A).

We next investigated whether the PI3K/Akt and MEK/ERK signaling pathways were involved in increasing the expression of cyclin D1. Because the phosphorylated forms of Akt and ERK 1/2 have been shown to be indirect indicators of the activities of their upstream signaling molecules PI3K and MEK, respectively, we determined the involvement of PI3K and MEK in the HBx-induced effects using a pharmacological approach to block their activities. After treating with LY294002, a PI3K inhibitor, or PD184352, a MEK inhibitor, for 24 h, HBx-induced activation of Akt and ERK, as well as up-regulation of cyclin D1 protein levels, were greatly suppressed compared with non-treated controls (Figure 4D). In fact, treatment with PD184352 or LY294002 inhibited cyclin D1 expression at both the mRNA and protein levels (Figure 4D,E). Notably, LY294002 treatment caused a stronger reduction in intracellular cyclin D1 protein expression than treatment with PD184352 (Figure 4F). These data indicate that HBx expression in oval cells activates ERK and Akt, leading to increased expression of cyclin D1 protein.

### 2.5. HBx Effects on Oval Cell Proliferation Are Abolished by LY294002 or PD184352

**Figure 5.**
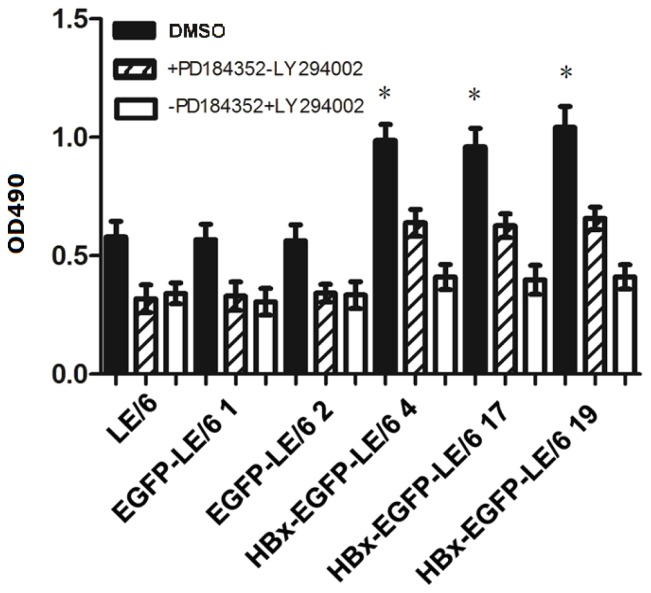
MTT assay of HBx-EGFP-LE/6, EGFP-LE/6, and LE/6 oval cell lines treated with PD184352 or LY294002. Six different cell lines, namely LE/6, EGFP1, EGFP2, HBx-EGFP4, HBx-EGFP17, and HBx-EGFP19, were exposed to PD184352 (5 μM), LY294002 (25 μM), or a vehicle control (DMSO) in media containing 10% FBS for 72 h. An MTT assay was performed and results showed that treatment with either PD184352 or LY294002 significantly inhibited HBx-induced oval cell proliferation. An asterisk * indicates *p* < 0.05 *vs.* vehicle control (DMSO). The data shown represent the mean ± SD of three independent experiments.

After confirming that overexpression of HBx activated the PI3K/Akt and MEK/ERK signaling pathways and promoted proliferation in oval cells, we examined whether activation of these pathways play a vital role in HBx-induced proliferation in oval cells. We found that treatment of oval cells with either LY294002 or PD184352 inhibited HBx-induced proliferation, as assessed by the MTT assay (Figure 5). These data indicated that both the PI-3K/Akt and ERK signaling pathways are required for HBx-induced proliferation in oval cells.

### 2.6. Discussion

Based on previous findings that oval cells proliferate during hepatic infection and that elevated HBx protein expression correlates with diseased liver tissues, we investigated whether HBx affected oval cell proliferation *in vitro.* Our results showed that overexpression of HBx indeed promoted oval cell proliferation and increased cyclin D1 expression at both the mRNA and protein levels.

A previous study initially suggested the proliferative role of HBx in hepatocytes [24]. In contrast, other research groups have reported that expression of HBx halts cell cycle progression, potentially via elevated levels of p21 and p27 proteins [25]. This discrepancy might be due to the fact that the two studies used different cells types that have distinct genetic backgrounds.

Cyclin D1 is widely believed to be able to regulate cell progression through the G1 phase of the cell cycle [26,27]. In this study, we found increased cyclin D1 expression in HBx-transfected oval cells, while HBx overexpression had no effect on p27, CDK2, or CDK4 protein expression. These results suggested that cyclin D1 might play a key role in HBx-induced oval cell proliferation. Importantly, HBx expression also led to a significant increase in cyclin D1 mRNA expression, indicating that the up-regulation of cyclin D1 protein expression was due to increased transcriptional activity in oval cells.

To gain further insights into the molecular mechanisms by which HBx induced cell proliferation and increased cyclin D1 expression in oval cells, we examined intracellular signaling pathways. HBx expression significantly increased phosphorylation of Akt and ERK when compared with non-transfected or mock-transfected controls. We also found that treatment with either PI3K or MEK inhibitors, LY294002 [28,29] or PD184352 [30,31], inhibited the activation of Akt and ERK in oval cells, indicating that phosphorylation of Akt and ERK depended on the activities of PI3K and MEK. We next examined the functional involvement of the PI3K/Akt and MEK/ERK signaling pathways in HBx-induced proliferation and cyclin D1 expression. Because treatment with either LY294002 or PD184352 inhibited HBx-induced proliferation and cyclin D1 expression in oval cells, we concluded that HBx-induced oval cell proliferation and up-regulation of cyclin D1 expression were dependent on the activation of PI3K/Akt and MEK/ERK signaling pathways. However, neither LY294002 nor PD184352 treatment completely abolished the HBx-induced cell proliferation, implying that there were alternative signaling pathways contributing to HBx-induced proliferation in oval cells. For example, up-regulation of cyclin D1 by HBx could be mediated by the nuclear factor-kappa B2/B-cell lymphoma 3 complex through the kappa B binding site on the cyclin D1 promoter [32].

Together, our results demonstrate that HBx promotes oval cell proliferation *in vitro*. These findings support the hypothesis that HBV-encoded proteins stimulate the proliferation of oval cells in the absence of an immune response. However, the effect of HBx on oval cell proliferation has not yet been determined *in vivo*. More research is needed to explore the relationship between HBx and oval cell proliferation *in vivo*.

## 3. Experimental Section

### 3.1. Cell Lines and Cell Culture

Hepatic oval cell lines (LE/6) were kindly provided by Prof. Nelson Fausto. The LE/6 cell line used in this study is comprised of non-tumorigenic cells that were derived from the liver of an adult rat fed a choline-deficient diet containing 0.1% ethionine for six weeks. These cells possess a high nuclear-to-cytoplasmic ratio and are organelle poor. Their morphological and biochemical properties have been previously characterized, which demonstrated that they have stem cell properties [10,33] HBx-EGFP-LE6 and EGFP-LE6 stable cell lines were established in our laboratory as previously reported [23]. Briefly, the purified *HBx* gene fragment was inserted into an enhanced green fluorescent protein N1 (pEGFP-N1) expression vector, and the recombinant plasmid pEGFP-HBx (p-HBx) was validated by double digestion with restriction endonuclease and DNA sequencing analysis. LE/6 cells were transfected by p-HBx or pEGFP-N1 using Lipofectamine 2000 transfection reagent (Invitrogen, Carlsbad, CA, USA) according to the manufacturer’s instructions. HBx-L02 cell lines were kindly provided by Dr. Shengli Yang in our lab [34]. These cells were maintained in a 1:1 mixture of DMEM (high glucose) (GIBCO, Carlsbad, CA, USA) and Ham’s F10 supplement (GIBCO, USA), which contains 10% heat-inactivated fetal bovine serum (FBS, GIBCO, USA), 1 μg/mL insulin (Sigma, Santa Clara, CA, USA), 0.5 μg/mL hydrocortisone (Sigma, USA), 25 μg/mL gentamicin (GIBCO, USA), and 250 μg/mL G418. When cells reached 80%–90% confluence, they were trypsinized and replated at a 1:3 split ratio. After every two to three passages, cells were stored in freezing medium (GIBCO, USA) and kept in liquid nitrogen until use.

### 3.2. Cell Proliferation Assay

Cell proliferation was measured by an MTT assay and a cell count assay. Briefly, a subconfluent monolayer of cells was trypsinized, and 1 × 10^3^ viable cells were suspended in 100 μL DMEM supplemented with 5% FBS. The cells were uniformly seeded in each well of 96-well plates and grown in DMEM supplemented with 5% FBS. After 24 h, the media was removed and replaced with fresh media. The plates were incubated at 37 °C in a humidified atmosphere of 5% CO_2_ for another 72 h. At indicated time-points, cells were suspended by trypsinization. For the cell count assay, the number of viable cells was counted in a hemocytometer using trypan blue. For the MTT assay, relative cell numbers were determined by incubating cells with MTT for 4 h. The resulting formazan was dissolved in DMSO, and A490 was measured in a 96-well plate-reader (Bio-Rad Company, Hercules, CA, USA). The absorbance at 490 nm is directly proportional to the number of viable cells. All experiments were performed three times.

In other experiments, cells were treated with the MEK inhibitor PD184352 (5 μM) or the PI3K inhibitor LY294002 (25 μM) for 72 h. Every 24 h, the media was replaced with fresh media and inhibitor. Relative cell numbers were measured by the MTT assay as described above. All experiments were performed three times.

### 3.3. Quantitative Real-Time PCR (qPCR) Analysis

Following reverse transcription, cDNA samples were diluted 1:5 in RNase-free water. The primers were designed according to the cDNA sequences in the GeneBank database using Primers Express software (Applied Biosystems, Foster City, CA, USA). Primer sequences were: *cyclin D1* forward 5′-GCGTACCCTGACACCAATCTC-3′ and reverse 5′-ACTTGAAGTAAGATACGGAGGGC-3′; *β-actin* forward 5′-GAAGTACCCCATTGAACACGG-3′ and reverse 5′-TTAGGGTTCAGAGGGGCC TC-3′. Each qPCR reaction was composed of SYBR Green mix, cDNA template, 10 μM forward primer, 10 μM reverse primer, and double distilled H_2_O in a total volume of 20 μL. Reactions were performed with a Real-Time PCR System 7500 (Applied Biosystems, Foster City, CA, USA). Reaction conditions were 94 °C denaturation for 1 min, followed by 40 cycles of denaturation at 94 °C for 60 s, annealing at 55 °C for 60 s, and extension at 72 °C for 60 s. The program was set to automatically record the average fluorescence value during the last 10% of time in each cycle, which is proportional to the amount of PCR product present at the end of each cycle. After reactions finished, the baseline and threshold were adjusted in the ABI 7500 software system (Applied Biosystems, Foster City, CA, USA) where the *C*t value of each reaction was determined. Data were analyzed with the comparative *C*t method and were normalized to β-actin expression in each sample. qPCR was performed in triplicate for each sample.

### 3.4. Western Blotting Analysis

Subconfluent cells were incubated overnight in the absence of serum and then treated with various compounds. The cells were lysed with ice-cold lysis buffer containing 50 mM Tris–HCl (pH 7.6), 150 mM NaCl, 0.1% sodium dodecyl sulfate, 1 mM dithiothreitol, 10 mM NaF, 2 mM Na_3_VO_4_, and 1× Complete Protease Inhibitor Cocktail (Roche Molecular Biochemicals, Ingelheim, Germany). Forty micrograms of each soluble protein sample were separated by 10% SDS-PAGE, transferred to nitrocellulose membranes using the Bio-Rad electrotransfer system (Bio-Rad laboratories, Richmond, CA, USA), blocked in 5× BSA/TBST (1× TBS and 0.1% Tween 20), and probed with each primary antibody overnight at 4 °C. After incubation with horseradish peroxidase-conjugated secondary antibody (1:4000), protein signals were detected using enhanced chemiluminescence (Amersham, Uppsala, Sweden). Equal protein loading was ensured by immunoblot of non-phosphorylated ERK and Akt, using rabbit polyclonal antibodies against the respective proteins. The primary antibodies used in this study were raised in mouse against HBx (RD Technology, Minneapolis, MN, USA), p27 (Cell Signaling Technology, Danvers, MA, USA), CDK2 (Santa Cruz Biotechnology, Santa Cruz, CA, USA), CDK4 (Santa Cruz Biotechnology), pERK (Cell Signaling Technology, USA), ERK (Cell Signaling Technology, USA), pAkt (Cell Signaling Technology, USA), Akt (Cell Signaling Technology, USA), pJNK (Cell Signaling Technology, USA), JNK (Cell Signaling Technology, USA), pp38 (Cell Signaling Technology, USA), p38 (Cell Signaling Technology, USA), and β-actin (Santa Cruz Biotechnology).

To study the association between MAPK signaling and increased cyclin D1 protein expression, cells were plated on 10 cm dishes and grown to 70% confluence. The growth media was replaced with fresh media with or without PD184352 or LY294002. At the end of the stimulation/treatment period, cells were harvested and cell lysates were prepared as described previously. Western blotting analysis was performed as described above, and densitometry of protein bands was determined by pixel intensity using Quantity One software (Bio-Rad Company, Hercules, CA, USA). All experiments were performed three times.

### 3.5. Statistical Analysis

All experiments were repeated at least three times. Data are presented as mean with SD. One-way ANOVA was used for multiple group comparisons. A *p*-value less than 0.05 was considered statistically significant.

## 4. Conclusions

Our results showed that overexpression of HBx promoted proliferation of oval cells and significantly up-regulated cyclin D1 expression in oval cells *in vitro*. We also found that HBx activated the PI-3K/Akt and MEK/ERK1/2 signaling pathways in oval cells. Furthermore, the HBx-induced increase of cyclin D1 expression and cell proliferation were almost completely abolished by treatment with either the MEK inhibitor PD184352 or the PI-3K inhibitor LY294002. In conclusion, these results indicated that HBx-induced oval cell proliferation and up-regulation of cyclin D1 protein expression were dependent on the activation of the MEK/ERK and PI3K/Akt signaling pathways.
